# Clinical profile and management of primary thyroid cancer in patients with nodular goitre at the Douala General Hospital, Cameroon

**DOI:** 10.11604/pamj.2021.38.405.25614

**Published:** 2021-04-28

**Authors:** Edgar Mandeng Ma Linwa, Esthelle Minka Ngom, Georges Enownchong Enow Orock, Charlotte Eposse Ekoube, Esther Eleonore Ngo Linwa, Ngenge Michael Budzi, Martin Geh Meh, Richard Njock Louis

**Affiliations:** 1 Faculty of Health Sciences, University of Buea, Buea, Cameroon,; 2 Faculty of Medicine and Pharmaceutical Sciences, University of Douala, Douala, Cameroon,; 3 Faculty of Health Sciences University of Bamenda, Bamenda, Cameroon,; 4 Faculty of Medicine and Biomedical Sciences University of Yaoundé I, Yaoundé, Cameroon

**Keywords:** Nodular goitre, thyroid, cancer, Cameroon

## Abstract

**Introduction:**

thyroid cancer (TC) is considered to have become the fastest growing cancer in terms of incidence worldwide. Despite literature reporting a prevalence of 5-10% in clinically identified thyroid nodules, Cameroon still has limited data on the profile of TCs in patients with Nodular Goitres (NGs). The Objective were to describe the epidemiological, diagnostic and therapeutic profiles of TCs in patients with nodular goitres at the Douala General Hospital (DGH).

**Methods:**

this was a retrospective cross-sectional analysis of patient records with diagnoses of NGs, over 11 years (2006 to 2016) at the DGH.

**Results:**

overall, 187 patients (mean age= 46.8±13.9 years, men=27 (14.4%)) were included; 43 (23%) cancers were identified. The most common histological type was papillary cancer (50%). Nodule size of >4cm and hypoechogenicity were independently associated with malignancy. Most patients presented with TNM stage II (47.4%) and well-differentiated cancers were considered to be predominantly at low-risk according to MACIS (55%) and AMES (74%) scores. Surgery was offered to 95.3% of patients.

**Conclusion:**

TCs are frequent in patients with NGs with papillary cancer dominating. A high index of suspicion should be held if a nodule is >4cm and/or is hypoechogenic. Prognostic studies are needed to describe the outcome of TCs in our setting.

## Introduction

A thyroid nodule is a discrete lesion within the thyroid gland that is radiologically distinct from the surrounding parenchyma [1]. Four to seven percent of the adult population has a palpable thyroid nodule [2]. The prevalence is much greater with the inclusion of nodules that are detected by ultrasonography (incidentalomas) or at autopsy. By the latter assessment, approximately 50 percent of 60-year-old persons have thyroid nodules [3]. Up to 5-20% of clinically identified nodules are malignant [4]. This corresponds to approximately 2 to 4 per 100,000 people per year, constituting only 1% of all cancers and 0.5% of all cancer deaths [5].

Thyroid cancer (TC) however, remains the most common malignant endocrine tumour worldwide and is considered to have become the fastest growing cancer in terms of incidence [3] and if recent trends are maintained, thyroid cancer may become the fourth most common cancer by 2030 in the United States [6]. In Saudi Arabia, it is the 2^nd^ most common cancer in women and the 4^th^ in men [7]. There are 5 main types of primary thyroid cancers: well differentiated (follicular and papillary), poorly differentiated (PDC), undifferentiated (anaplastic) and the sporadic cancers (medullary cancer). The classic treatment for TC is conventional thyroidectomy with adjuvant radioiodine ablation, and most patients can be cured with these treatments.

In Cameroon, thyroid incidentalomas were found to be very frequent with a prevalence of 28.3% and potential risk of malignancy in 12.9% [8]. Literature has demonstrated that mortality is higher in regions of endemic goitre because of more frequent advanced tumor stages at diagnosis and an increased ratio of more aggressive subtypes [9]. The Cameroon National Programme for the prevention and control of Iodine Deficiency was initiated in 1991 and was reported to be successful in reducing Iodine Deficiency Disorders [10]. However, few studies are available in our setting which determine the extent of thyroid nodules at presentation and demonstrate the characteristics that are associated with thyroid cancer. We therefore conducted this study with the following objectives: 1) determine the frequency and distribution of thyroid cancers in patients with nodular goitres; 2) compare nodular and clinical characteristics between benign and malignant nodular goitres; 3) compare the prognostic characteristics by age and gender in patients with thyroid cancers and 4. describe the management of patients with thyroid cancers.

## Methods

**Study design, period and setting**: this was a hospital-based retrospective cross-sectional study at the ENT, Oncology/Radiotherapy, Pathology, and Endocrinology units of the Douala General Hospital (DGH) which is a third-level referral hospital in the Littoral Region of Cameroon. Records of patients were reviewed over a period of 11 years (2006 to 2016).

**Participants and sampling**: the study population was made up of patients who presented with a nodular goitre at any of the above units between 1^st^ of January 2006 and 31^st^ of December 2016. We included records of patients with sonographic and histopathological reports and excluded those diagnosed out of the study period, with incomplete records, inconclusive biopsy and/or secondary/metastatic cancers.

**Study procedures and variables**: data was collected using a pre-tested questionnaire. Clinical variables collected were age at diagnosis, gender, presence of palpable cervical lymph nodes. Nodular variables included size of thyroid nodule (in cm), echogenicity and location of nodule (on ultrasound). All considered predictor variables. Prognostic variables included the American Joint Cancer Committee (AJCC), Tumour Node Metastasis (TNM) stage, Metastasis Age at diagnosis Completeness if resection , local Invasion, Size of tumour (MACIS) and Age at diagnosis, Metastasis, Extent of tumour, Size of tumour (AMES) scores.

**Data sources and definitions**: only patients´ records were reviewed. Nodule size was considered as the largest diameter of a thyroid nodule as reported by the ultrasound report. In case of multinodular goitre, the size of the largest nodule evaluated was considered. Inconclusive biopsy was regarded as any biopsy result (apart from Bethesda II) that was not confirmed with excisional biopsy. Any record without age and sex was considered incomplete. Low-risk cancer was considered when MACIS score was less than 6. Patients´ records that lacked at least a report on cervical lymph node status and a chest X-ray or other metastatic work-up were considered not stageable.

**Sample size considerations**: using a pre-study prevalence estimate from South Africa (11,1%) [2], the minimum sample size (N) as determined by the Cochran´s formula,

$$N=\frac{Z^{2}p(1-p)}{e^{2}}$$

Where Z is the confidence level at 95% with a standard value 1.96, e is the allowable error of 5%, while p is the estimated prevalence according to a similar study of 11.1%, was 152 participants.

**Data management and data analysis**: the data collected was entered into and analyzed using SPSS version 20 statistical software. Quantitative variables were represented by their mean, standard deviation and range. Qualitative variables were represented as frequencies and percentages. Categorical variables were compared using the Chi-square or Fisher´s exact tests. The Student´s T-test was used to compare means. The level of statistical significance was set at p < 0.05.

**Ethical considerations**: ethical approval was granted by the Institutional Review Board of the Faculty of Health Sciences of the University of Buea. Administrative approval was obtained from the Director of the Douala General Hospital. Data entry forms were coded to ensure anonymity of patient personal information.

## Results

**Socio-demographic characteristics of the study population**: the ages of the patients at presentation ranged between 15 and 85 years with a mean age of 46.8 ± 13.9 years. The male to female ratio was 1: 5.9.

**Figure 1:**
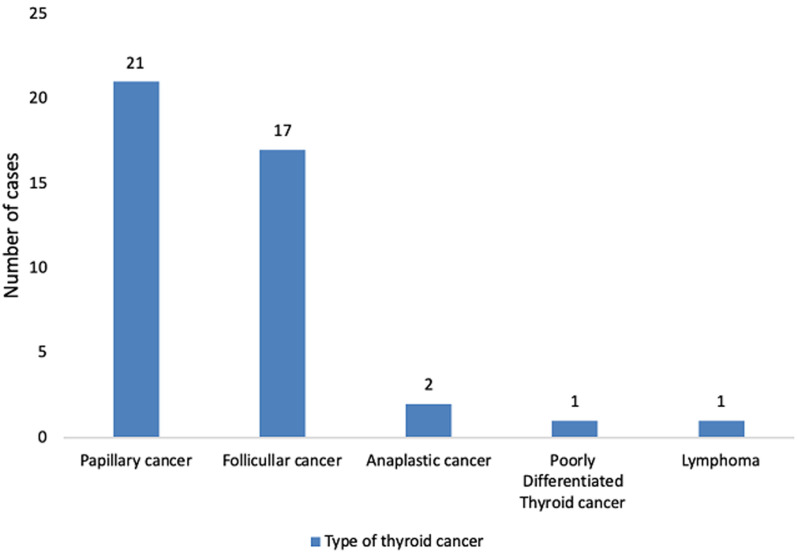
types of thyroid cancer

**Frequency and distribution of thyroid cancers in patients with nodular goitres**: twenty three percent (n=43) of nodular goitres were malignant. Papillary cancer was the most common histological entity (50.0%) and this was followed by follicular cancer (40.5%) as shown in Figure 1.

**Comparison of nodular and clinical characteristics between benign and malignant nodular goitres**: the presence of cervical lymph nodes, nodule size >4cm, solitary thyroid nodules and hypoechogenicity were characteristics associated with malignancy as shown in Table 1. On multivariate analyses, using logistic regressions, we observed that hypoechogenicity (aOR= 5.6, 95% CI 1.3-23.9 p= 0.020) and nodule size >4cm (aOR= 23.2, 95% CI 4.6-177.2 p<0.001) were the only two factors independently associated with thyroid cancer. The details of these associations are shown in Table 2.

**Table 1:** nodular characteristics in malignant and benign nodular goitres

Characteristics	Type of nodular goitre, n(%)		P-value
	Benign N=144	Malignant N=43	
**Age at diagnosis**			
<45 (n=80)	60 (41.5)	20(46.5)	
>45 (n=107)	8(58.5)	23(53.5)	0.573NS
Gender			
Male (n=27)	17 (11.8)	10 (23.3)	
Female (n=160)	127(88.2)	33(76.7)	0.061NS
**Palpable cervical lymphnodesa**			
Yes (n=12)	3(4.4)	9(23.1)	
No(n=96)	66(95.6)	30(76.9)	0.003S
Nodule size (cm)			
< 4 (n=133)	118(81.9)	15(34.9)	
> 4 (n= 54)	26(18.1)	28(65.1)	<0.001
**Nodularity**			
Multinodular Goitre (n=36)	34(23.6)	2(4.7)	
Single Thyroid Nodule (n=151)	110(76.4)	41(95.3)	0.006
**Echogenicity**			
Hypoechogenic nodule (n=56)	28(19.4)	28(65.1)	
Hyper/Isoechogenic nodule(n=131)	116(80.5)	15(34.9)	<0.001

a=data for only 108 patients was available. NS=Not significant. S=Significant

**Table 2:** multivariate regression analysis of retrospective data for independent risk factors for malignancy of thyroid nodular goitres at Douala General Hospital (N=188)

Characteristics	aOR	95% CI	P-value
Nodule >4cm	23.2	4.6-177.2	<0.001
Hypoechogenicity	5.6	1.3-23.9	0.020
Solitary thyroid nodule	9.6	0.9-100.7	0.059
Presence of cervical lymph node	11.2	1.0-127.5	0.052

aOR=adjusted odds ratio; CI=Confidence Interval

**Comparison of the prognostic characteristics by age and gender in patients with thyroid cancers**: follicular cancer was found to be the most common cancer in males, whereas, papillary cancer was the most frequent in females. Papillary cancers were more common in patients above 45 years whereas follicular cancers predominated in patients <45 years. One in every two females presented with Stage II disease. Patients > 45 years predominantly presented with Stage II while non-stageable disease was the most common in younger patients. Majority of patients were categorised as low-risk based on AMES and MACIS scores. All males were low-risk while upto 30.4% of the females were high risk according to AMES scores. Based on age, 62.5% and 37.5% of patients above 45years were low-risk based on AMES and MACIS scores respectively.

**Management of patients with thyroid cancers**: the most commonly practiced surgical treatment for males (80%) was total thyroidectomy only. It was however offered to only a few females (51.6%). Total thyroidectomy plus neck dissection was done in 20% of male versus 16.2% female patients. Thus, the most extensive surgical procedures were offered more to males. Total thyroidectomy plus neck dissection was offered in greater proportion (57.1% versus 42.9%) to patients > 45years. More males and patients aged < 45 years received chemotherapy and external beam radiotherapy. The observed differences were not statistically significant.

## Discussion

In this hospital-based study of medical records from 2006 to 2016, we found that one in five adults with nodular goitres had thyroid cancer. The majority were papillary cancers and factors associated with cancer were presence of palpable cervical lymph nodes, nodule size >4cm, solitary thyroid nodules and hypoechogenicity. Based on risk stratification systems, most patients presented with cancers that are considered low-risk cancers. In terms of management, the majority had total thyroidectomy alone offered.

Cancer prevalence as high as 38% has been reported in India, although regions like to Madagascar (22.3%) and Iran (28%) report prevalence similar to our study [11,12]. Though our study had a higher prevalence than that obtained in South Africa (11.1%) [2] the explanation may lie in the fact that, the latter study recruited all thyroid samples as opposed to only thyroid nodules as in our study. The most common histological type was papillary cancer as opposed to older studies [13,14] who found follicular cancer to be the most prevalent. This can be explained by the fact that these earlier studies were carried out in a context where most African countries were still considered iodine deficient areas. The distribution in our study correlates with recent studies carried out in Africa [15,16] and attests to the fact that Africa is gradually becoming an iodine sufficient area.

Though patients aged <45 years in our sample had a higher prevalence of malignancy, this was not statistically significant, as also demonstrated in studies from Korea and Brazil [17,18]. However, an association between malignancy, age <45 years [19] and age > 45 years [20] has been reported in other studies. The lack of association in our study can be explained by the much larger sample size used in the latter studies. Males are considered to be at higher risk of malignancy than females [2,20-22]. However, in agreement with few studies, [2,22] our study found no gender disparities. This may be explained by the relatively low frequency of males in our study. Despite the use of different evaluation methods [23-25], our study concurred with other studies in demonstrating an association between the presence of cervical lymph nodes and malignancy. Hypoechogenicity was present in up to 50% of patients and it was associated with malignancy. Other studies from Korea and Italy [17,26] denote a much lower prevalence of hypoechogenicity and refute any association with malignancy. This can be explained by the fact that these studies evaluated smaller nodules which predominantly display microcalcifications on ultrasound. Though literature [17,21] argues otherwise, nodules > 4cm were associated with malignancy and this in concordance with few hospital based studies conducted in Turkey and Brazil [18,23]. Our findings may be explained by our large mean nodule size of 4.13 cm. While some studies advocate MNG carry a higher risk of malignancy [18], others attribute the risk to sialyl-Thomsen-nouvelle (STN) [26]. In our study, STN was significantly associated with malignancy. Other studies however, refute any association [27,28]. These discrepancies can be explained by the few MNGs in our sample. On multivariate analysis, only nodule size and hypoechogenicity were independently associated with thyroid cancer.

While some studies have demonstrated that the more aggressive histologic subtypes have similar gender distribution [29], other studies have shown that men had a significantly higher proportion of aggressive tumours [30]. The latter findings were consistent with our study. Some studies report papillary cancer incidence to decrease with age whereas for all other sub-types, incidence rates generally increase with age. This is in discordance with our findings where the most aggressive subtypes predominated in patients <45 years, though these differences were not statistically significant. Studies report AJCC stage I or II disease to be the most common stage at presentation, findings similar to our study though with higher proportions than ours [31]. This signals a poorer prognosis in our setting and may be explained by earlier detection of cancers at screening. In our study, up to 74% and 55% of thyroid cancers were classified as low-risk according to AMES and MACIS scores respectively. This is lower than reports in western literature where more than 80.0% were low-risk on both risk stratification system [31,32] and this varied with age, contrary to our study.

Total thyroidectomy is the gold standard for patients with a preoperative diagnosis of papillary thyroid cancer when the nodule is greater than 1 cm in size. It is generally agreed upon that a therapeutic neck dissection should be pursued in the setting of well-differentiated thyroid cancer patients with clinically positive lymph nodes, whether in the central or lateral neck compartments [33]. In our study, total thyroidectomy only was the most commonly offered treatment modality and this management did not vary by age or gender. Our study had a number of limitations which must be mentioned. This was a single centre hospital based study which likely reflects the situation of the study hospital and so cannot be generalized to entire Cameroonian population. It was difficult to access all information from the records, so many records were excluded. The risk stratification scores used have neither been validated in our setting nor extensively worldwide. Finally, our sample size was small and hence might have failed to unearth certain associations or differences. However, this study is the first of its kind from Cameroon to explore and describe the clinical profiles of thyroid cancers in patients with nodular goiters in Cameroon, and hence will serve as a benchmark for future studies.

## Conclusion

Thyroid cancers are frequent in patients with nodular goitres in our setting with papillary cancer being the most common subtype. Most patients present with low-risk cancers based on TNM, AMES and MACIS scores. The most common management is surgical. A high index of suspicion should be considered in patients with nodules more than 4cm and/or which are hypoechogenic. There is need for further research to be conducted in order to understand the risk factors, prognostic characteristics and outcome of thyroid cancers in our setting.

### What is known about this topic

- In Cameroon, thyroid nodules have been shown to be frequent (28.3%) and 1/10 have potential risk of malignancy;
- In regions where goitre is endemic, mortality is higher and people consult at advanced tumor stages and have more aggressive subtypes. Ultimately, the clinical profile, cancer subtypes and prevalence of thyroid cancers depend on the prevalence of goitre in the population;
- After initiation of the iodization programme in Cameroun in 1991, few studies have been published to enable determine the impact of such programme on the profile and prevalence of thyroid cancers. Moreover, clinical characteristics that can enable practitioners rapidly identifying nodules with high risk of malignancy have not been determined.

### What this study adds

- The prevalence of thyroid cancers amongst patients with thyroid nodules was found to be 23%;
- In this paper, we show that some clinical characteristics of thyroid nodules ( nodules more than 4cm and hypoechogenicity) give them a higher probability of being malignant. This is significant because it will help clinicians to have a higher index suspicion for cancer especially when considering settings where all histopathological workups are not always available and this will ultimately fasten management;
- The clinical profile of thyroid nodules described and the cancer subtypes found in this study could be a telltale sign of the effective prevention and control of Iodine deficiency disorders.
